# Associations between the *MDM2* promoter P1 polymorphism del1518 (rs3730485) and incidence of cancer of the breast, lung, colon and prostate

**DOI:** 10.18632/oncotarget.8705

**Published:** 2016-04-12

**Authors:** Liv B. Gansmo, Lars Vatten, Pål Romundstad, Kristian Hveem, Bríd M. Ryan, Curtis C. Harris, Stian Knappskog, Per E. Lønning

**Affiliations:** ^1^ Section of Oncology, Department of Clinical Science, University of Bergen, Bergen, Norway; ^2^ Department of Oncology, Haukeland University Hospital, Bergen, Norway; ^3^ Department of Public Health, Faculty of Medicine, Norwegian University of Science and Technology, Trondheim, Norway; ^4^ Laboratory of Human Carcinogenesis, Center for Cancer Research, National Cancer Institute, Bethesda, MD, USA

**Keywords:** MDM2, polymorphism, del1518, SNP309, cancer risk

## Abstract

The *MDM2* promoter region contains several polymorphisms, some of which have been associated with *MDM2* expression, cancer risk and age at cancer onset. del1518 (rs3730485) is an indel polymorphism residing in the *MDM2* promoter P1 and is in almost complete linkage disequilibrium with the *MDM2* promoter P2 polymorphism SNP309T>G (rs2279744). Cancer risk assessments of del1518 have previously been conducted in relatively small Chinese populations only. In this study we assessed the genotype distribution of del1518 among healthy Caucasians, African Americans and Chinese, and we estimated the Odds Ratios (OR) for incident cancer of the breast, colon, lung and prostate (n=7,081) as compared to controls (n=3,749) in a large Caucasian (Norwegian) cohort.

We found the genotypes of the del1518 to vary significantly between healthy Caucasians, African-Americans and Chinese (p< 1×10^−5^). Further, we found a positive association of the del1518 del-allele with risk of colon cancer (dominant model: OR = 1.15; 95 % CI = 1.01 – 1.31). Stratifying according to SNP309 status, this association remained among carriers of the SNP309TG genotype (OR = 1.21; 95 % CI = 1.01 – 1.46), but with no clear association among carriers of the SNP309TT genotype. In conclusion, our findings suggest del1518 to be associated with increased risk of colon cancer.

## INTRODUCTION

The Mouse Double Minute 2 homolog (MDM2) is a major regulator of the tumor suppressor p53. The levels of MDM2 and p53 are tightly regulated, as they act in a negative feedback loop where p53 induces *MDM2* transcription in response to genotoxic stress, whereas MDM2 binds, inhibits and directs p53 for proteasomal degradation by ubiquitinylation [1–4]. MDM2 hyperactivity through mechanisms such as gene amplification, increased transcription and enhanced translation is observed in many human cancers harboring wild-type *TP53* [5–7], and *MDM2* overexpression has been suggested to act as an alternative mechanism of p53 inactivation, thus promoting tumor growth.

While multiple single nucleotide polymorphisms (SNPs) have been identified in *MDM2* promoter regions, so far, only a few have been assessed with respect to potential biological functions. SNP309T>G; rs2279744, located in the *MDM2* promoter P2, has been shown to increase *MDM2* expression by increasing the binding affinity between the promoter and the transcription factor Sp1 [8]. The same investigators found the SNP309G allele to be associated with early onset of several malignancies among individuals carrying *TP53* germline mutations (Li-Fraumeni syndrome) and also lower age at diagnosis of estrogen receptor (ER) rich breast cancer among individuals with wild-type *TP53* [9, 8]. Subsequent findings, with respect to the influence of SNP309 status on age at cancer onset as well as cancer risk have, however, been conflicting [10–17].

Recently, we reported a second *MDM2* promoter P2 polymorphism, SNP285G>C; rs117039649 [18]. SNP285 is in complete linkage disequilibrium (LD) with SNP309; thus forming a distinct SNP285C/309G haplotype [18, 19]. The SNP285C allele diminish Sp1 binding to the *MDM2* promoter, and has been found to be associated with reduced risk for breast, ovarian and endometrial cancer [18, 20–22], but was not associated with prostate or lung cancer risk [20, 21, 23].

del1518 (rs3730485), is an insertion/deletion polymorphism of 40 bps in the *MDM2* promoter P1 [24, 25]. The del1518 del-allele has been shown to reduce transcription from the *MDM2* P1 promoter in some cell lines [26] and LD analysis has shown that the del1518 locus has a high LD with the SNP309 locus [24]. Some small studies have assessed the potential effects of the del1518-variant on cancer risk, and reported that the del1518 del allele may be associated with an increased risk for hepatocellular carcinoma [27] but to be unrelated to risk for several other cancer forms, including cancer of the lung [24], breast [25] ovary [28] or esophagus [29] in Chinese populations.

In the present case-control study we compared del1518 distribution across ethnic cohorts (Caucasians, African Americans and Han Chinese) and assessed the association of del1518 status with the risk for breast-, lung-, colon- and prostate cancer.

## RESULTS

*MDM2* del1518 was determined in 7,081 Norwegian patients diagnosed with incidental cancers of the breast, colon, prostate or lung as well as 3,749 age-matched healthy individuals. In addition, to assess potential ethnic differences in del1518 distribution, we analyzed a cohort of 300 healthy African-Americans, and performed data mining from previously published del1518 genotyping of Chinese individuals (including 2,594 Chinese samples) [24, 25, 27–29].

### Distribution of del1518

**Figure 1:**
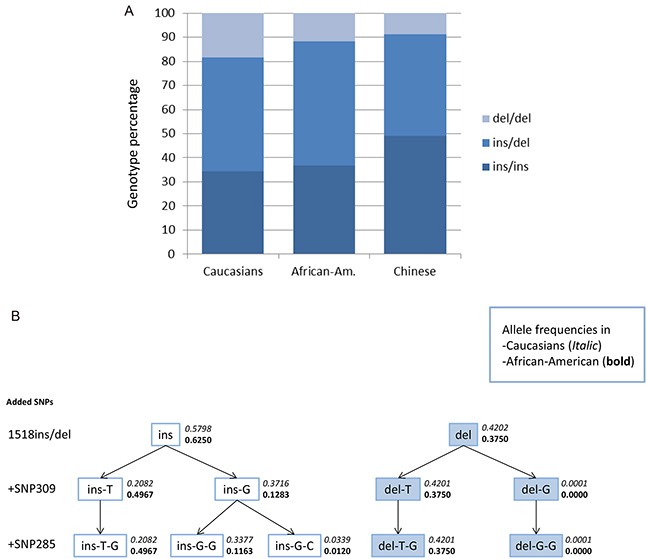
Genotype distribution and haplotype-tree **A.** Genotype distribution of *MDM2* SNP del1518 among Caucasians, African Americans and Chinese (Chinese data extracted from previous publications [24, 25, 27–29]). **B.** Haplotype-tree based on the three *MDM2* SNPs; del1518 (rs3730485), SNP309 (rs2279744) and SNP285 (rs117039649). The tree and the frequencies given for each haplotype are based on 3,749 healthy controls in the CONOR cohort and 300 healthy African Americans.

Among the 3,749 healthy Caucasian (Norwegian) individuals, we found the MAF of del1518 to be 0.42 and the genotype distribution to be in Hardy-Weinberg equilibrium (*p* = 0.5). Interestingly, this differed (*p* = 0.015) from the distribution observed in African Americans (MAF = 0.38). It also differed (*p* < 1×10^−5^) from the distribution previously reported in Chinese populations (MAF = 0.30) [24, 25, 27–29] (comparison of genotype distribution between all three populations, p < 1×10^−5^; Figure 1A).

Except for five individuals harboring the del1518 del/del – SNP309TG genotype and two individuals harboring the del1518 ins/del – SNP309GG (in the Caucasian cohort), the del1518 del variant was restricted to individuals carrying the SNP309TT or SNP309TG genotype, and del1518 del/del homozygosity was only seen among individuals carrying the SNP309TT genotype (Table 1; Figure 1B). Thus, we observed a very strong linkage disequilibrium (LD) between the del1518 variant and SNP309 (D'=0.999 for the healthy controls and D' = 0.997 for the entire sample set, including cancer patients). This observation is in accordance with previous findings in a Chinese population [24].

**Table 1:** Distribution of *MDM2* del1518 – SNP309 haplotypes (alleles)

Haplotype del1518 - 309	Healthy controls		Breast cancer		Colon cancer		Lung cancer		Prostate cancer	
	N	%	n	%	n	%	n	%	n	%
**ins – T**	1561	20,82	675	19,66	646	21,08	572	21,49	1058	21,15
**ins – G**	2786	37,16	1296	37,74	1085	35,41	946	35,54	1854	3707
**del – T**	3150	42,01	1463	42,60	1331	43,44	1143	42,94	2087	41,72
**del – G**	1	0,01	0	0	2	0,007	1	0,04	3	0,06
**Total**	7498	100	3434	100	3064	100	2662	100	5002	100

### *MDM2* del1518 and cancer risk

**Figure 2:**
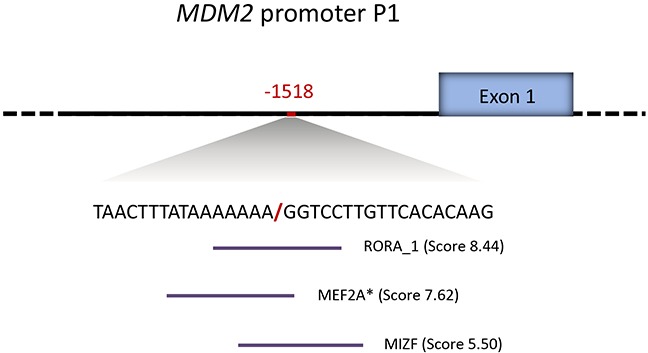
*MDM2* promoter P1 del1518 break-point sequence context Novel transcription factor binding sites generated by the *MDM2* del1518 del allele, as predicted by the JASPAR database (indicated by horizontal lines under the sequence). The predicted binding scores are annotated in the brackets. * indicates binding sites located on the reverse strand.

In order to assess the potential impact of del1518-status on cancer risk, we first performed *in silico* analyses of putative de novo transcription factor binding sites created by the deletion. Applying the prediction algorithm in the JASPAR database [30] with default settings (cut off threshold of 80%), we found the breakpoint of the del-variant, with flanking nucleotides, to generate new binding sites for RORA (RAR-Related Orphan Receptor A), MEF2A (Myocyte Enhancer Factor 2A) and MIZF (MBD2 (methyl-CpG-binding protein)-interacting zinc finger protein) (Figure 2).

To determine the potential correlation between *MDM2* del1518 status and cancer risk, we analyzed the distribution of this variant in large sample sets of incident cases of four major cancer forms (1,717 breast, 1,532 colon, 1,331 lung and 2,501 prostate cancer samples) drawn from the population-based Cohort of Norway (CONOR) [31] and compared the distribution to 3,749 matched controls without any cancer from the same cohort. The genotype distributions and the results from calculations of Odds Ratios (OR) between patients and controls are summarized in Table 2.

**Table 2:** *MDM2* del1518 distribution and cancer risk (OR)

Cases/controls	Genotype del1518 n (%)			OR (95% CI) del1518	Fisher exact	OR (95% CI) del1518	Fisher exact
	ins/ins	ins/del	del/del	Dominant model^a^		Recessive model^b^	
**Healthy Controls**	1285 (34.3)	1777 (47.4)	687 (18.3)	1.00	-	1.00	-
Women	636 (34.0)	877 (46.9)	359 (19.2)	1.00	-	1.00	-
Men	649 (34.6)	900 (47.9)	328 (17.5)	1.00	-	1.00	-
**Colon cancer**	478 (31.2)	775 (50.6)	279 (18.2)	1.15 (1.01-1.31)	0.034	0.99 (0.85-1.16)	0.938
Women	237 (30.4)	393 (50.5)	149 (19.1)	1.18 (0.98-1.41)	0.077	1.00 (0.81-1.23)	1.000
Men	241 (32.0)	382 (50.7)	130 (17.3)	1.12 (0.94-1.35)	0.218	0.99 (0.79-1.23)	0.955
Left side	190 (30.2)	331 (52.5)	109 (17.3)	1.21 (1.01-1.45)	0.045	0.93 (0.75-1.17)	0.577
Right side	261 (31.5)	410 (49.5)	158 (19.1)	1.14 (0.97-1.33)	0.133	1.05 (0.87-1.27)	0.621
**Lung cancer**	447 (33.6)	624 (46.9)	260 (19.5)	1.03 (0.90-1.18)	0.662	1.08 (0.92-1.27)	0.346
Women	155 (31.2)	247 (49.7)	95 (19.1)	1.14 (0.92-1.40)	0.261	1.00 (0.78-1.28)	1.000
Men	292 (35.0)	377 (45.2)	165 (19.8)	0.98 (0.83-1.16)	0.827	1.17 (0.95-1.43)	0.161
**Breast cancer^c^**	581 (33.8)	809 (47.1)	327 (19.0)	1.01 (0.88-1.16)	0.944	0.99 (0.84-1.17)	0.932
**Prostate cancer^d^**	836 (33.4)	1240 (49.6)	425 (17.0)	1.05 (0.93-1.19)	0.439	1.02 (0.85-1.22)	0.853

a del/del + ins/del versus ins/ins

b del/del versus ins/del + ins/ins

c compared to female controls only

d compared to male controls only

**Figure 3:**
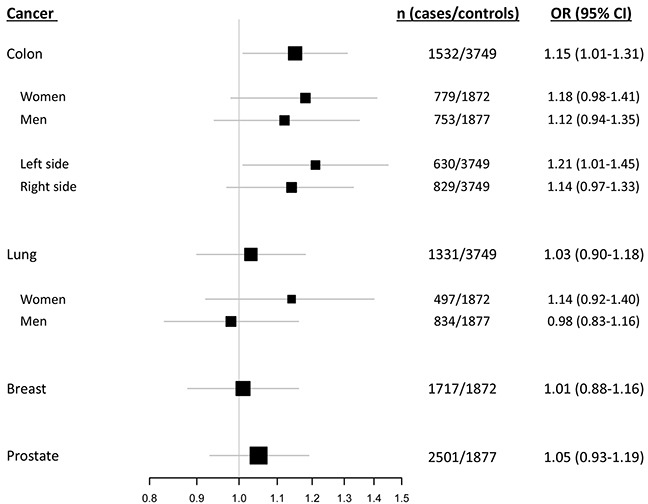
del1518 polymorphism and cancer risk Forest plot illustrating the effect of the *MDM2* del1518 polymorphism on the risk of breast-, colon-, lung- and prostate cancer, applying the dominant model (comparison of del1518 del/del + del/ins genotypes versus the ins/ins genotype).

In overall assessments applying a dominant model, the del1518 del allele was associated with an increased risk of colon cancer (OR = 1.15; 95 % CI = 1.01 – 1.31; Table 2, Figure 3, Supplementary Table S1). Notably, this association was more pronounced among patients with left sided colon cancer (dominant model: OR = 1.21; 95 % CI = 1.01 - 1.45), compared to patients with right sided tumors (dominant model: OR = 1.14; 95 % CI = 0.97 – 1.33; Table 2, Figure 3, Supplementary Table S1). For the three other cancer forms, no statistically significant association between del1518 status and cancer risk was recorded (Table 2, Figure 3, Supplementary Table S1).

### Potential interactions between *MDM2* del1518 status and *MDM2* SNP309

**Figure 4:**
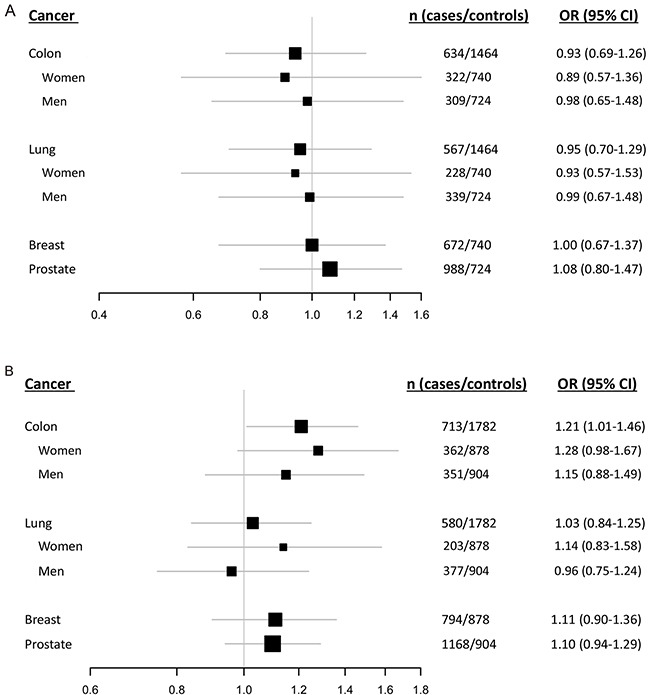
Impact of the del1518 polymorphism on cancer risk among SNP309 Forest plots illustrating the effect of the *MDM2* del1518 polymorphism on the risk of breast-, colon-, lung- and prostate cancer, applying the dominant model (comparison of del1518 del/del + del/ins genotypes versus the ins/ins genotype) among individuals carrying the SNP309TT genotype **A.** and (del1518 del/ins versus ins/ins) among individuals harboring the SNP309TG genotype **B.** (Due to the strong linkage between the del1518 del-allele and the SNP309T-allele, the group of homozygous del/del was non-evaluable within individuals with the SNP309TG-genotype).

In the samples investigated in this study we have previously reported the *MDM2* SNP309GG/TG genotypes to be associated with reduced risk of lung cancer among women, but not associated with risk of breast-, colon- or prostate cancer [20]. Given that the del1518 del was strongly linked to the SNP309T allele, we further refined our analyses by removing individuals harboring the SNP309GG genotype and assessed the effect of del1518 separately in individuals harboring the SNP309TG or SNP309TT genotype. Among individuals carrying the SNP309TT genotype, no association was recorded in either analysis (Table 3A, Figure 4A). However, among individuals with the SNP309TG genotype, the del1518 del allele was associated with increased risk for colon cancer (OR = 1.21; 95 % CI = 1.01 – 1.46; Table 3B, Figure 4B), indicating a possible synergism between the del1518 del-allele and the SNP309G-allele.

**Table 3A:** *MDM2* del1518 among SNP309TT

Cases/controls	Genotype del1518 n (%)			OR (95% CI) del1518	Fisher exact	OR (95% CI) del1518	Fisher exact
	ins/ins	ins/del	del/del	Dominant model^a^		Recessive model^b^	
**Healthy Controls**	155 (10.6)	623 (42.6)	686 (46.9)	1.00	-	1.00	
Women	70 (12.3)	311 (41.1)	359 (46.6)	1.00	-	1.00	
Men	85 (10.2)	312 (42.9)	327 (46.9)	1.00	-	1.00	
**Colon cancer**	71 (11.3)	283 (44.9)	277 (43.9)	0.93 (0.69-1.26)	0.646	0.89 (0.74-1.07)	0.958
Women	34 (10.6)	140 (43.5)	148 (46.0)	0.89 (0.57-1.36)	0.576	0.90 (0.69-1.17)	0.463
Men	37 (12.0)	142 (46.3)	129 (4.8)	0.98 (0.65-1.48)	0.916	0.87 (0.67-1.14)	0.338
**Lung cancer**	63 (11.1)	245 (43.2)	259 (45.7)	0.95 (0.70-1.29)	0.749	0.95 (0.79-1.16)	0.655
Women	23 (10.1)	110 (48.3)	95 (41.7)	0.93 (0.57-1.53)	0.797	0.76 (0.56-1.02)	0.081
Men	40 (11.8)	135 (39.8)	164 (48.4)	0.99 (0.67-1.48)	1.000	1.14 (0.88-1.47)	0.355
**Breast cancer^c^**	66 (9.8)	279 (41.5)	327 (48.7)	1.00 (0.67-1.37)	0.857	1.01 (0.82-1.24)	0.958
**Prostate cancer^d^**	108 (10.9)	456 (46.2)	424 (42.9)	1.08 (0.80-1.47)	0.643	0.91 (0.75-1.11)	0.375

a del/del + ins/del versus ins/ins

b del/del versus ins/del + ins/ins

c compared to female controls only

d compared to male controls only

**Table 3B:** *MDM2* del1518 among SNP309TG

Cases/controls	Genotype del1518 n (%)			OR (95% CI) del1518	Fisher exact
	ins/ins	ins/del	del/del^a^	Ins/del vs. ins/ins	
**Healthy Controls**	628 (35.2)	1154 (64.8)	-	1.00	-
Women	312 (35.5)	566 (64.5)			
Men	316 (35.0)	588 (65.0)			
**Colon cancer**	221 (31.0)	492 (69.0)	-	1.21 (1.01-1.46)	0.044
Women	109 (30.1)	253 (69.9)		1.28 (0.98-1.67)	0.075
Men	112 (31.9)	239 (68.1)		1.15 (0.88-1.49)	0.320
**Lung cancer**	201 (34.7)	379 (65.3)	-	1.03 (0.84-1.25)	0.841
Women	66 (32.5)	137 (67.5)		1.14 (0.83-1.58)	0.462
Men	135 (35.8)	242 (64.2)		0.96 (0.75-1.24)	0.797
**Breast cancer^b^**	264 (33.3)	530 (66.8)	-	1.11 (0.90-1.36)	0.328
**Prostate cancer^c^**	386 (33.1)	782 (67.0)	-	1.10 (0.94-1.29)	0.234

a no observations since del1518 del is linked to SNP309T

b compared to female controls only

c compared to male controls only

Similar subgroup analyses of the three remaining cancer forms showed no association between del1518 status and risk for malignancy at any site (Table 3A–3B, Figure 4A–4B).

## DISCUSSION

The findings by Levine's group [8] and our group [18], suggesting that both the *MDM2* promoter SNP's 309 and 285 may have an effect on cancer risk, implies the importance of fine tuning of MDM2 levels with respect to carcinogenesis, and also indicate that other *MDM2* promoter variants could potentially be of importance.

Assessments of the promoter P1 del1518 variant have previously been performed in a few relatively small Chinese cohorts [24, 25, 27–29]. In the present study, we determined the distribution of del1518 status in a large Caucasian cohort. A major strength of our study is the large number of cancer patients and controls compared to other studies assessing del1518-status with respect to cancer risk. Also, the population based design, using incident cancers (breast, lung, colon and prostate) for whom DNA samples were collected years prior to diagnosis and controls matched by gender, age, and residential area, is a strong feature.

In line with previous risk assessments in Chinese populations [24, 25, 28], we found no association between del1518 status and risk for cancer of the breast, lung or prostate. We did, however, observe a positive association with colon cancer risk. Somewhat surprisingly, we found an effect on colon cancer among individuals heterozygous, but not homozygous, for del1518. However, as the del1518 variant is linked to the SNP309T allele, homozygosity for del1518 is restricted to individuals harboring the SNP309TT genotype. Additionally, after stratifying our data according to SNP309 status, we found heterozygousity for the del1518 del allele to be associated with an increased risk of colon cancer among individuals carrying the SNP309TG genotype, but not among SNP309TT homozygous individuals. Although the statistical significance was borderline, this may indicate a synergistic effect of these two polymorphisms. Notably, studies on other genes have revealed that individuals harboring heterozygous mutations may be at risk of different diseases compared to homozygous carriers [32, 33]. Such effects may potentially differ between cancer forms as well; previously, we reported that the SNP285C allele significantly reduces the risk for breast cancer among SNP309GG homozygous individuals, whereas in ovarian cancer, the effect of SNP285C was significant among SNP309TG individuals only [20, 18]. Here, the possibility exists that del1518 may be detrimental in concert with SNP309G heterozygosity (individuals harboring the SNP309TG genotype), but not exert a similar effect among SNP309TT carriers.

Left sided and right sided colon cancers are known to be different with respect to histological and molecular characteristics [34], and also the genetic mechanisms initiating the tumorigenic process have been found to be dissimilar [35]. There are evidence that hereditary nonpolyposis colon cancer (HNPCC) in general are found in right sided colon cancer [36], while familial adenomatous polyposis (FAP) dominates in left sided colon cancer [37]. However, although we only observed an increased OR among individuals suffering from left sided colon cancer, a clear trend for increased risk was observed also among patients harboring right sided colon cancer. The lack of statistical significance in this group may be a result of limited statistical power. Thus, whether the del1518 polymorphism is associated with increased risk for both left sided – and right sided colon cancer may need to be explored in independent populations.

Previously, we showed that *MDM2* SNP285 is part of a predicted estrogen response element (ERE), where both Sp1 and ERα may bind and affect tumorigenesis [21]. However, when assessing SNP285 and cancer risk among “gender neutral” cancer forms (i.e., lung and colon cancer), we observed only moderate gender differences [20]. In the present study, corresponding to our previous findings regarding SNP285, we found no gender specific effect of del1518 on cancer risk in either cancer form. Taken together, these data indicate that the effect on cancer risk of the germline variations in the *MDM2* promoter regions, in general, are more dependent on tissue type than on gender.

In conclusion, we find an association of del1518 with the risk of colorectal cancer and no association for cancer of the prostate, breast or lung. The finding for colon cancer, however, warrants confirmation by others.

## MATERIALS AND METHODS

### Study populations

To assess potential impact of del1518 status on cancer risk, we genotyped this variant in blood DNA from 10,830 individuals drawn from the Norwegian population-based Cohort of Norway (CONOR) study [31], previously described in [20]. This sample set included 3,749 cancer-free controls as well as incident cases of four major cancer forms: cancer of the breast (n=1,717), prostate (n=2,501), lung (n=1,331) and colon (n=1,532).

The African-American cohort (n=300) was from the Laboratory of Human Carcinogenesis, Center for Cancer Research, National Cancer Institute Bethesda, USA. These samples were collected as part of an ongoing case control study with previously described inclusion criteria [38]. Briefly, population controls were identified from the Department of Motor Vehicles, MD, USA and frequency matched to cases by age and gender. Written informed consent was obtained from all participants, and the study was approved by the Institutional Review Boards of the participating institutions.

### *In silico* predictions

Potential novel binding sites for transcription factors, generated by the deletion variant of the *MDM2* del1518 polymorphism, were predicted using the JASPAR database [30] (http://jaspardev.genereg.net). Predictions were made using the default settings of the database (cut off threshold of 80%).

### *MDM2* del1518 genotyping

All samples were genotyped for *MDM2* del1518 by using DNA extracted from peripheral white blood cells. The region of the *MDM2* promoter P1 containing the del1518 was amplified by PCR using the VWR Taq DNA Polymerase system (VWR), with a primer pair previously described [27]. The amplification was performed in a total reaction volume of 25 μl, containing 2.5 μl 10x Key Buffer, 0.2 mM dNTPs, 0.2 μM each primer, 1.25 U Taq polymerase and 1 μl template DNA (10-100 μg). The thermo-cycling conditions were an initial step of 94°C for 5 min, 35 cycles of 94°C for 30 sec, 58°C for 30 sec and 72°C for 30 sec, followed by a final elongation step of 72°C for 10 min. The PCR products were separated by electrophoresis in a 3% agarose gel and visualized by GelRed^TM^ Nucleic Acid Gel Stain (BIOTIUM). The del1518 insertion and deletion alleles were observed as 481 bps – and 441 bps bands respectively.

All samples were previously genotyped for *MDM2* SNP285 and SNP309 status [20] using custom LightSNiP assays (TIB MOLBIOL Syntheselabor GmbH, Berlin, Germany) as described in detail elsewhere [39].

### Statistical analysis

Potential deviations from Hardy-Weinberg equilibrium in cancer patients as well as healthy controls were evaluated using the Chi-square test. Possible associations between *MDM2* del1518 and cancer risk, both in total and in stratified groups, were estimated by Odds Ratios (OR) and Fisher exact tests. ORs are given with 95% confidence intervals (CI) and p-values from Fisher exact tests are given as two-sided and cumulative.

All statistical analyses were performed using the IBM SPSS statistics (version 19) software package.

## SUPPLEMENTARY TABLES


