# Supplementary material for: Associations between the MDM2 promoter P1 polymorphism del1518 (rs3730485) and incidence of cancer of the breast, lung, colon and prostate
Source: Oncotarget. 2016 Apr 12;7(19):28637–46. doi: 10.18632/oncotarget.8705 (PMC5053751; doi:10.18632/oncotarget.8705)
Supplement: Supplementary file 1 [file oncotarget-07-28637-s001.pdf]

SUPPLEMENTARY TABLE

Supplementary Table S1: *MDM2* del1518

Cases/controls	Genotype del1518 n (%)			OR (95% CI) del1518	Fisher exact	OR (95% CI) del1518	Fisher exact
	ins/ins	ins/del	del/del	del/del vs. ins/ins		Ins/del vs. ins/ins	
**Healthy Controls**	155 (10.6)	623 (42.6)	686 (46.9)	1.00	-	1.00	
Women	70 (12.3)	311 (41.1)	359 (46.6)	1.00	-	1.00	
Men	85 (10.2)	312 (42.9)	327 (46.9)	1.00	-	1.00	
**Colon cancer**	71 (11.3)	283 (44.9)	277 (43.9)	1.09 (0.92-1.30)	0.326	1.17 (1.03-1.34)	0.022
Women	34 (10.6)	140 (43.5)	148 (46.0)	1.11 (0.87-1.42)	0.385	1.20 (0.99-1.46)	0.060
Men	37 (12.0)	142 (46.3)	129 (4.8)	1.07 (0.83-1.37)	0.607	1.14 (0.95-1.38)	0.177
**Lung cancer**	63 (11.1)	245 (43.2)	259 (45.7)	1.09 (0.91-1.30)	0.359	1.01 (0.88-1.16)	0.914
Women	23 (10.1)	110 (48.3)	95 (41.7)	1.09 (0.82-1.45)	0.607	1.16 (0.92-1.45)	0.211
Men	40 (11.8)	135 (39.8)	164 (48.4)	1.12 (0.89-1.41)	0.371	0.93 (0.78-1.12)	0.454
**Breast cancer**[a]	66 (9.8)	279 (41.5)	327 (48.7)	1.00 (0.83-1.20)	1.000	1.01 (0.87-1.17)	0.910
**Prostate cancer**[b]	108 (10.9)	456 (46.2)	424 (42.9)	1.06 (0.84-1.20)	0.964	1.07 (0.94-1.22)	0.339

[a]female controls only
[b]male controls only
